# Compositional and microhardness findings in tooth affected by X-linked hypophosphatemic rickets

**DOI:** 10.4317/jced.56945

**Published:** 2020-07-01

**Authors:** Francisco-Samuel-Rodrigues Carvalho, Victor-Pinheiro Feitosa, Cristiane-Sá Roriz Fonteles, Thyciana-Rodrigues Ribeiro, Bruno-Sousa Araújo, Alejandro-Pedro Ayala, Fábio-Wildson-Gurgel Costa

**Affiliations:** 1 DDS, MSc. Division of Oral and Maxillofacial Surgery, Federal University of Ceará Campus Sobral, Sobral, Ceará, Brazil; 2 DDS, PhD. Research Division, Paulo Picanço School of Dentistry, Fortaleza, Brazil; 3 DDS, PhD. Post-graduate Program in Dentistry, School of Dentistry, Federal University of Ceará, Fortaleza, Ceará, Brazil; 4 MSc. Division of Physics, Federal University of Ceará, Fortaleza, Ceará, Brazil; 5 PhD. Division of Physics, Federal University of Ceará, Fortaleza, Ceará, Brazil

## Abstract

**Background:**

This study aimed to evaluate the X-linked hypophosphatemic rickets (XLHR)-related compositional and microhardness tooth aspects.

**Material and Methods:**

One affected and one non-affected teeth by XLHR were sectioned transversely, and each section was separated for Micro-Raman spectroscopy, Knoop microhardness and scanning electron microscopy with energy dispersive x-ray microanalysis (SEM-EDS). The outcomes of these analyses were assessed.

**Results:**

Outcomes of Raman analysis of inorganic/organic components (~958/~1250+~1450 cm−1) and carbonate/phosphate (~1070/~958 cm−1) ratios showed areas of altered enamel and dentin (interglobular dentin, calcospherites, and mantle dentin) with an increase of inorganic content in the rickets tooth. Microhardness reduction was observed in the affected tooth, with a more evident drop in regions of mantle dentin, interglobular dentin, and calcospherites. SEM-EDS analysis showed demonstrated the absence of calcium and phosphorus in interglobular spaces.

**Conclusions:**

In conclusion, compositional and structural deficiencies were observed in deciduous tooth affected by XLHR. Also, it was observed the absence of hydroxyapatite in the interglobular dentin by using Raman spectroscopy analysis.

** Key words:**Dentin, dentin permeability, X-linked hypophosphatemic rickets, tooth, tooth calcification, Raman spectroscopy.

## Introduction

Familial hypophosphatemic rickets is a rare condition (1-3), and it could be transmitted as a X-linked inheritance, depicting mutation in gene of phosphate regulation (PHEX) located at chromosome Xp22.1 (3,4), predominantly expressed in osteoblasts and odontoblasts (3). Absence or reduction of such gene’s activity is correlated to the augment of phosphatonin fibroblast growth factor 23 (FGF23) expression, which promotes renal phosphate wasting (5). Due to this disturb on phosphate metabolism, X-linked hypophosphatemic rickets (XLHR) may depict alterations both in skeleton (1,6) and in oral environment with structural issues such as loss of mineralization in dentin, periapical lesions, early tooth loss, and spontaneous abscess (1,7).

Early injuries in teeth of patients with rickets are believed to be associated with structural alterations on enamel and dentin, thereby favoring bacterial invasion of dentin-pulp complex (8). These patients usually present micro-fractures in enamel (8), presence of wide interglobular spaces (3), increased porosity in dentin nearby pulpal chamber and disorganized dentin matrix (3,6). In a recent study with micro-computed tomography (MicroCT), teeth from patients with XLHR showed severe structural alteration of dentin, with marked presence of interglobular dentin (1). However, this investigation did not undertake ultra-structural analysis to survey the deposition of each component. In another study (3) which used SEM, no significant modifications in calcium/phosphate ratio were observed, although structural defects were noted principally in interglobular spaces. SEM has been employed in several investigations (2,9-13). However, particularly in teeth affected by XLHR, a comprehensive analysis of hydroxyapatite and collagen distribution in dentin is desirable along with the investigation of possible alterations of chemical bonds, what is not feasible to be obtained by using SEM-EDS micro-analysis.

In this regard, micro-Raman spectroscopy (MRS) might be a valuable analytic technique able to measure the chemical composition and bonds of biological complexes like biofluids, cells, and tissues, including bone and teeth. Besides, MRS may attain molecular fingerprint of different substrated, providing quantitative information of chemical composition (14,15). Therefore, Raman analysis might detect biochemical changes in molecular level, thereby achieving reliable usage for diagnosis, survey of new therapies (14), and characterization of different dental substrates (16,17). Further advantages of MRS in comparison with other photonic devices are the performance and speed of diagnostic.

Acquisition in MRS is performed by the geometry of back-scattering with the new for light transmission through the specimen. It is useful in particular for *in vivo* diagnosis and assay of thick tissues. Moreover, the employment of visible light reduces the effects of water absorption, allowing measures in body fluids or cells in liquid environments (14). Raman spectroscopy represents a modality of non-invasive, chemically selective analysis to generate non-destructive images with valuable composition information in 1 µm resolution (15). Despite these several advantages of Raman spectroscopy, to our knowledge, no reports regarding the investigation of teeth from patients with XLHR by means of MRS alone or in combination with SEM, EDS or Knoop microhardness experiments.

Thus, the present investigation aimed to assess ultra-structure, organic matrix and mineral distribution as well the microhardness of different areas in teeth from patients with XLHR by using aforementioned analyses.

## Material and Methods

-Samples 

This *in vitro* study evaluated two primary mandibular molar extracted teeth from two children (a XLHR affected male and his non-affected sister) previously assayed in a micro computed tomography investigation (1). They were stored in 0.1% thymol solution until analyses.

-Micro-Raman Spectroscopy

Raman micro-spectrophotometer (Xplora, Horiba, Paris, France) was used to survey dental substrates in three areas of sound dentin, enamel, and altered dentin (calcospherites, interglobular spaces, and mantle dentin). Spatial distribution of organic and inorganic compounds was determined by relative intensities of Raman peaks (16,17). He-Ne laser was employed with 638nm wavelength and 3.2 mW power focused on 10X and 100X optical lenses (Olympus). Spectra were obtained in the range 400-4000 cm-1 with 10s acquisition and 3 accumulations.

Peaks assessed were: 1) mineral/organic matrix ratio, which was determined by the intensity of ʋ1 phosphate band vibration (~958 cm−1) divided by the combination of intensities of proline and hydroxyproline bands (~1250 + ~1450 cm−1); 2) carbonate/phosphate ratio was measured by intensities of carbonate (~1070 cm−1) and ʋ1 phosphate (~958 cm−1) (18). We adopted herein the relation between intensities due to the variability in acquisition in Raman micro-spectroscopy (10,18,19).

A Raman map was also obtained in LabRAM HR (Horiba) using diode laser (785 nm), operating at 50 mW power, with 0.5 µm laser scanning area. All acquisitions were attained in same conditions using approximately 1 µm laser penetrations in samples. Total areas of around 100X100 µm2 were scanned with 100X magnification and, approximately, 5x5 µm2 macropixels using a DuoScan module. The Raman spectra were firstly acquired in LabSpec 5 software (Horiba Jobin Yvon Inc, NJ, USA).

Knoop Microhardness

Transversal sections of each tooth were polished with 400-, 500- and 600-grit SiC papers under water irrigation. Polishing clothes with diamond pastes were used afterward. Between and after final polishing steps, specimens were ultrasonically cleaned with saline for 3 minutes (20).

Specimens (n=5) from each tooth were then positioned in a microhardness tester HMV-2000 (Shimadzu Corporation, Kyoto, Japan) with a Knoop indenter. Microhardness was surveyed with 10g for 15s. Indentation points were at enamel and dentin areas. Mean Knoop hardness of each hard tissue were obtained.

-SEM-EDS analysis

Further transversal section was polished as aforementioned, dehydrated and gold sputter coated with QT150ES (Quorum Technologies Ltd., Lewes, East Sussex, UK) for SEM observation. Inspect 50 microscope (FEI, Amsterdam, Netherlands) was used equipped with an EDX device. Altogether, these procedures allowed the assessment of surface and compositional alterations of specimens.

## Results

-Micro-Raman Spectroscopy

Outcomes of Raman analysis of inorganic/organic matrices (~958/~1250+~1450 cm−1) and carbonate/phosphate (~1070/~958 cm−1) ratios depicted higher preservation of inorganic/organic ratio in enamel than in dentin both in control tooth and in the tooth from a patient with rickets. Areas of altered dentin (interglobular dentin, calcospherites, and mantle dentin) showed increased inorganic ratio in comparison with other areas of same specimen with apparent unaffected dentin (Table 1). Carbon/phosphate ratio presented inversion of normal pattern for tooth affected with rickets in relation to enamel/dentin of normal tooth, in all areas surveyed (Table 1).

**Table 1:**
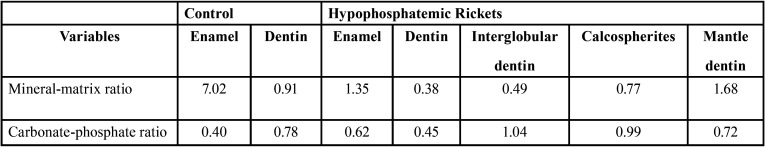
Raman peak ratio of different dental substrates.

**Figure 1:**
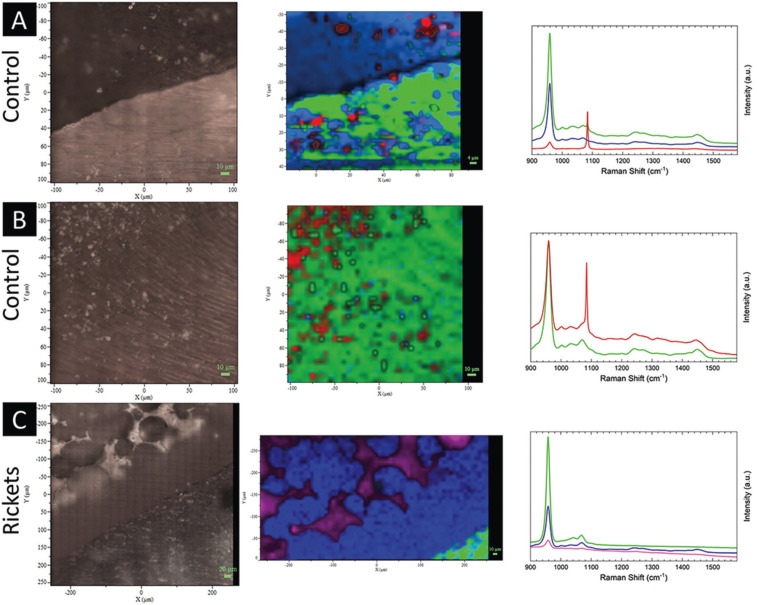
Evaluation of the dental substrate through Raman spectroscopy with a range of 800 cm-1 to 1600 cm-1. The relative intensities are based on the hydroxyapatite ~960 cm-1 (green represents enamel, and blue represents dentin), and carbonate ~1070 cm-1 (red) peaks and applied to the tooth with rickets. At dentin-enamel transition zone (A) and dentin region (B) of the control tooth, there is homogeneity in the substrate. The dentin enamel transition region in rickets tooth shows heterogeneity in the dentin substrate (C); also, it is possible to observe the absence of hydroxyapatite peak between the calcospherites as highlighted in purple color.

**Figure 2:**
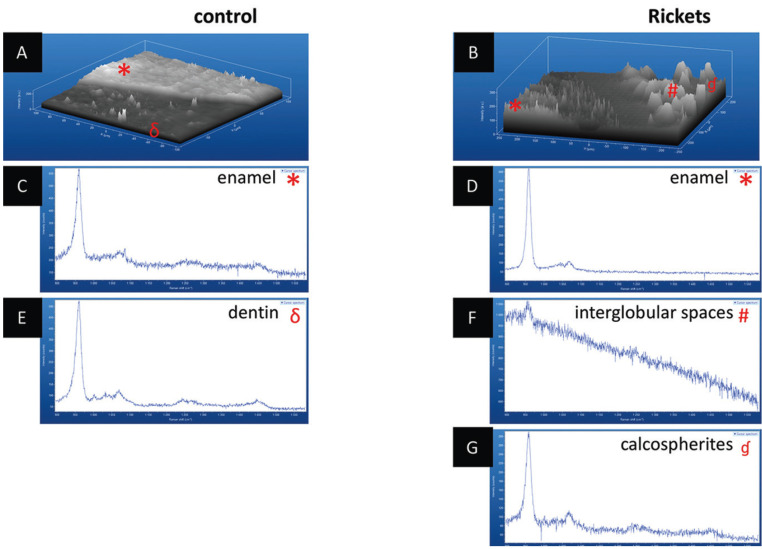
Evaluation of enamel-dentin zone through 3D Raman spectroscopy. A) dentin enamel transition region in the control tooth showing homogeneity in both portions of the substrate. B) enamel-dentin transition region in the tooth with rickets showing heterogeneity in dentin substrate. C) enamel Raman spectra of the control tooth. D) enamel Raman spectra of the tooth with rickets. E) Raman spectra of the tooth control dentin. F) Raman spectra of the interglobular spaces. G) Raman spectra of the calcospherites.

Raman analysis in 3D images highlighted the relative intensity of phosphate in the dentin-enamel junction (DEJ). Higher concentration of phosphate was found in enamel, sound dentin and in calcospherite, while interglobular dentin depicted the absence of hydroxyapatite peak (Figs. 1,2).

-Knoop Microhardness

The structural strength of teeth was assayed by microhardness assessment using a Knoop indenter at enamel, DEJ, dentin, and altered dentin. Diminishing of microhardness was observed in teeth from patients with hypophosphatemic rickets, with a more evident drop in regions of mantle dentin, interglobular dentin, and calcospherites ( Table 2).

**Table 2:**
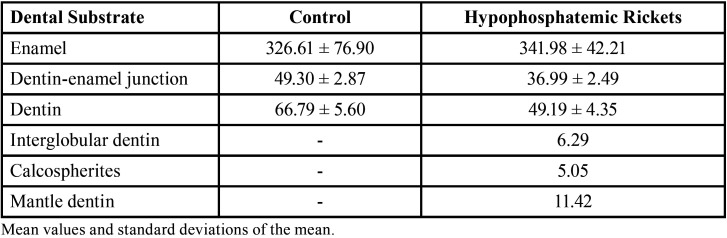
Knoop microhardness in different areas of the calcified dental substrates.

-SEM-EDS

**Figure 3:**
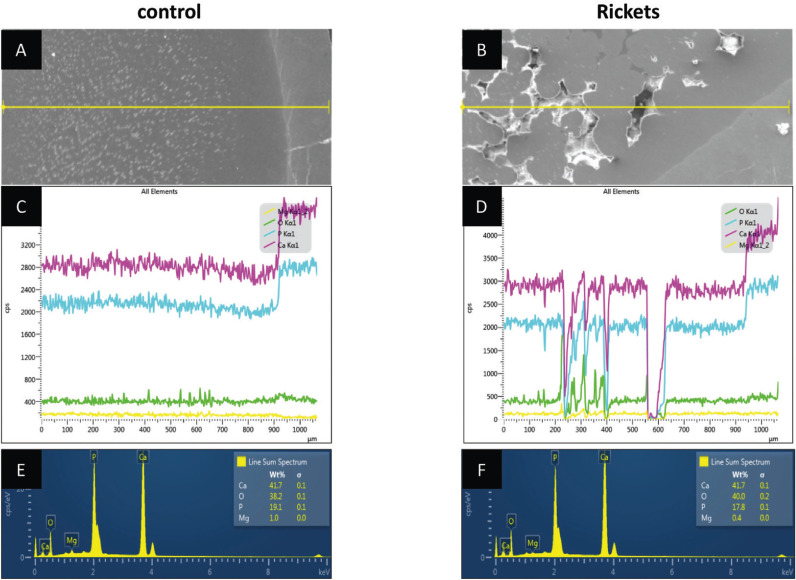
Energy-dispersive X-ray spectroscopy for evaluation of calcium, phosphorus, and magnesium concentrations. A: transverse section of the control tooth showing enamel and dentin. B: transverse section of the rickets tooth showing enamel and dentin. C: Graph of the image A composition showing homogeneity of its structural composition with higher values observed in the enamel. D: Graph of the image B composition showing heterogeneity of the interglobular dentin and calcospherite structural compositions and presenting higher values in the enamel. E: Graph representing the analyzed field in image A and its evaluated ions percentage rate. F: Graph representing the analyzed field in image B and its evaluated ions percentage rate.

Enamel and dentin near DEJ did not depict remarkable differences in SEM and EDS (Fig. 3). However, dentin area close to pulpal chamber presented striking alterations in XLHR-related tooth, with the presence of large interglobular spaces among calcospherites, with minor changes in EDS, except for calcium and phosphorus alteration. It is possible to note heterogeneity of calcospherites distribution along dentin near pulpal chamber, with coalescence patterns and a variety of sizes of calcospherites.

EDS analysis, overall, did not show altered calcium-phosphorus concentrations in control (sound) teeth (Ca 41.7%, *P* 19.1%; Ca/P ratio: 2.18) and in teeth from patients with rickets (Ca 41.7%, *P* 17.8%; Ca/P ratio: 2.34). Nevertheless, the image of substance concentration demonstrated absence of calcium and phosphorus in interglobular spaces. This finding was confirmed by linear acquisition (Fig. 3).

## Discussion

The dentin structure knowledge is an important aspect due to its association with genetic disorders that affect the mineralization process, as observed in XLHR (13). Dental manifestations of this form of rickets have been directly related to the dentinal structural deformity, which promotes repercussion on the other components of the dental organ (1,3,7). These findings occur due to abnormalities in the circumumpulpar dentin mineralization associated with failure in the calcospherites mineralization process. Inadequate coalescence of these globules of calcification results in an irregular calcification pattern. These aspects justify, in part, the findings observed in the present study, especially the presence of calcospherites surrounded by an expressive amount of interglobular dentin. In fact, this finding was also supported by other authors who have found similar results (13,21).

The development of dentin, enamel, and cement usually involves remarkable coordination of molecular complexes and cellular events that culminate in the formation of united tissues at distinct interfaces (16). These structures consist of organic and inorganic components, mainly amide and hydroxyapatite, respectively (22). Patients with rickets have structural deformities in the deciduous dentition, mainly in the dentin due to changes in the proportions of calcium and phosphorus, as well as changes in the crystallinity of the hydroxyapatite (23). These findings are supported by the hypothesis that nucleation and growth of hydroxyapatite crystals are dependent on the action of non-collagenous proteins abundantly present in dentin, especially proteins known as Small Integrin Binding Ligand N-linked Glycoproteins (SIBLINGs) (11,16-18,23,24). The Raman spectroscopy analysis performed in the present research did not identify characteristic peaks of any of these SIBLING proteins probably due to overlapping peaks generated in this evaluation and the absence of Raman spectroscopy studies that have characterized pure samples of these non-collagenous proteins to date. However, we believe the fact of SIBLING proteins play key roles in the development of the hydroxyapatite crystals may justify the findings observed in the present study.

Raman spectroscopy was presently used to characterize the dentin of individuals with XLHR because it is a rapid and advanced analytical technique that allows determining the structural and chemical composition of several molecules, providing information based on the vibrational mode of chemical bonds (16).

The present study evaluated the inorganic/organic matrix (~958/~1250 + ~1450 cm−1) and carbonate/phosphate (~1070/~958 cm−1) ratios because they determine the quality of the hydroxyapatite (18). In healthy teeth, it has been described that this quality is higher in enamel than in dentin (16,22,25). Thus, this finding highlights the importance of investigating such Raman spectra in teeth affected by XLHR. Raman microspectroscopy suggests that the ratio of the inorganic/organic contents of the dentin is smaller than that of the enamel, similarly to dentine in relation to cement (16,17). This information was observed in the present study both by the findings of the Raman microspectroscopy and by the reduction of the dentin Knoop microhardness in relation to the enamel of the tooth with XLHR.

As previously described by other authors (3,11,21,26), a large amount of interglobular dentin has been observed in the deciduous teeth affected by rickets. This type of dentin is characterized as a hypomineralized tissue occurring in several pathological conditions (e. g, rickets, biliary atresia, and fluorosis), and is distributed among mineralized globular masses (27). Presently, analysis of the interglobular dentin, as well as mantle dentin and calcospherites regions showed important changes of their Raman spectra and reduced resistance based on the obtained values of Knoop microhardness, demonstrating important structural fragility. Also, significant dentin alterations in proximity to the pulp chamber were observed in the present study, which is a well-known finding in the literature (1,3,13). In fact, structural abnormalities in the morphology of different dentin layers and failure in the mineralization process (12) are probably correlated with the observed clinical manifestations in teeth of patients with rickets, especially their reduced longevity. In this context, Soares *et al* (7) have reported recurrent spontaneous abscess development and a significant number of missing teeth of patients affected by XLHR, including the individuals from which teeth were analyzed in this paper.

In this study, three different regions of dentin were submitted to the microhardness test. Interglobular dentin and calcospherites were the two regions with the lowest values of microhardness. Probably it is an expected finding because of the heterogeneity in dentin surface as well as the size of the microindentation device (17). The heterogeneity in the dentin affected by XLHR may justify the fact that calcospherite presented a lower microhardness value than the interglobular dentin. By the way, scanning electron microscopy showed calcospherites with different arrangements, degrees of coalescence, and sizes, similarly to that described by Seeto *et al.* (10).

Calcospherites are uniformly observed under normal conditions and can be originated from growth centers. However, the impairment in their coalescence process favors the formation of interglobular spaces, which are characteristic features in hypophosphatemic rickets (3). Such changes culminate with the dentin morphology as observed in computed tomography (1). Histological and immunohistochemical studies have also described similar features and correlate their presence with structural fragility (3,28). In the present investigation, calcospherites heterogeneity regarding arrangement and coalescence patterns justify the different microhardness results despite of the different degrees of mineralization observed in dentin adjacent to the pulp chamber.

The present SEM-EDS analysis demonstrated findings commonly reported in XLHR studies, including abnormalities in dentin close to pulp chamber and absence of significant alterations in predentin and dentin close to dentinoenamel junction (3,11-13).

This study showed compositional and structural deficiencies in deciduous tooth affected by XLHR, remarkable in regions composed by calchospherites, interglobular dentin, and mantle dentin. Also, the absence of hydroxyapatite in the interglobular dentin by using Raman spectroscopy analysis was a relevant finding added to the current literature, and it should be considered in further studies evaluating other clinical forms of rickets.
